# Alcohol Pretreatment to Eliminate the Interference of Micro Additive Particles in the Identification of Microplastics Using Raman Spectroscopy

**DOI:** 10.1021/acs.est.2c01551

**Published:** 2022-08-25

**Authors:** Dunzhu Li, Emmet D. Sheerin, Yunhong Shi, Liwen Xiao, Luming Yang, John J. Boland, Jing Jing Wang

**Affiliations:** † AMBER Research Centre and Centre for Research on Adaptive Nanostructures and Nanodevices (CRANN), Trinity College Dublin, Dublin D02 PN40, Ireland; ‡ Department of Civil, Structural and Environmental Engineering, Trinity College Dublin, Dublin D02 PN40, Ireland; § TrinityHaus, Trinity College Dublin, Dublin D02 PN40, Ireland; ∥ School of Chemistry, Trinity College Dublin, Dublin D02 PN40, Ireland

**Keywords:** microplastic, microadditive particles, Raman spectroscopy, alcohol pretreatment, hit quality index

## Abstract

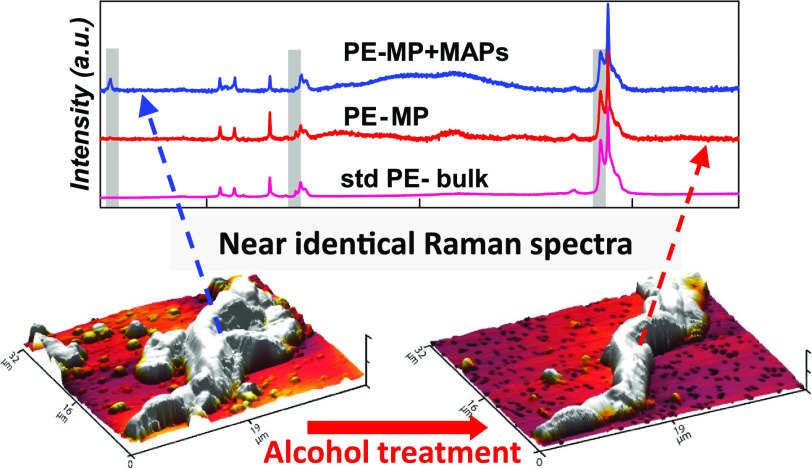

Raman spectroscopy is an indispensable tool in the analysis of microplastics smaller than 20 μm. However, due to its limitation, Raman spectroscopy may be incapable of effectively distinguishing microplastics from micro additive particles. To validate this hypothesis, we characterized and compared the Raman spectra of six typical slip additives with polyethylene and found that their hit quality index values (0.93–0.96) are much higher than the accepted threshold value (0.70) used to identify microplastics. To prevent this interference, a new protocol involving an alcohol treatment step was introduced to successfully eliminate additive particles and accurately identify microplastics. Tests using the new protocol showed that three typical plastic products (polyethylene pellets, polyethylene bottle caps, and polypropylene food containers) can simultaneously release microplastic-like additive particles and microplastics regardless of the plastic type, daily-use scenario, or service duration. Micro additive particles can also adsorb onto and modify the surfaces of microplastics in a manner that may potentially increase their health risks. This study not only reveals the hidden problem associated with the substantial interference of additive particles in microplastic detection but also provides a cost-effective method to eliminate this interference and a rigorous basis to quantify the risks associated with microplastic exposure.

## Abstract 2

Alcohol pretreatment eliminates the potential misidentification of the chemical additives and microplastic particles released during plastic degradation by enabling particle separation and individual analysis.

## 1 Introduction

Microplastics (MPs, solid-polymer-containing particles, European Chemical Agency^1^) are a growing global concern,^2−4^ especially MPs <10 μm in size because they can translocate from the gut cavity to the lymph and circulatory systems, causing systemic exposure and accumulation in the tissues of humans and animals.^5^ Currently, Raman spectroscopy is an indispensable tool^6−11^ to identify and characterize the chemical composition of single MP particles below 20 μm, including nanosized MP down to 50 nm^10^ (NMPs, <1000 nm^12,13^). Raman spectroscopy is nondestructive, highly accurate, and generates spectra due to the interaction of light with local bond vibrations.^6^ Despite these advantages, it is incapable of distinguishing between materials with subtle differences in the chemical structure, since some fingerprint vibrations such as the carbonyl group (C=O) are weakly detected.^14^

However, distinguishing between target substances and reference materials with similar chemical structures is essential in MP studies. Modern plastics are a complex cocktail of polymers, chemical additives, and residual monomers.^15^ On average, nonfiber plastics contain 93% polymer resin and 7% additives by mass.^16^ Typical organic additives consist of small molecules, which are substantially different from the polymers (macromolecules) comprised of repeating monomer units. The potential risks of these additives heavily depend on their physicochemical properties. For instance, the additive butylated hydroxytoluene (BHT) can primarily target the liver, increasing liver weight and enzyme activity,^17^ while the additive erucamide has low toxicity to human health.^18^ While polymer-based MPs <10 μm can translocate and accumulate in tissues, such as the liver and kidneys, the specific risk of MPs to human health is still unknown.^5^ For these reasons, it is crucial to separately determine the levels of additives and MPs for accurate exposure assessments and effective management of their potential risks.

Many additives are insoluble in water while some (i.e., slip additives) are designed to naturally migrate to the surface of plastics.^19,20^ Consequently, the latter additives are likely to be released into water, especially during the initial stages of plastic degradation. More importantly, some additives have a chemical structure very similar to that of their parent polymers. For example, behenamide (CH_3_(CH_2_)_20_CONH_2_) is a typical slip additive widely used in polyethylene (PE) plastic (i.e., water bottle caps^21,22^). It comprises a long saturated alkyl chain terminated at one end by an amide group (i.e., adjoining carbonyl and amine groups). The PE polymer, on the other hand, is comprised exclusively of long saturated alkyl chains. Given the strong Raman signal associated with these saturated alkyl chains (i.e., ν(C–H)) and relatively weak Raman signals from carbonyl and amine groups,^14,23^ it is extremely difficult to distinguish between pure micro additive particles (MAPs) derived from a slip additive such as behenamide and PE MPs from the parent plastic.

It is well known in food safety studies that plastic packaging is a significant source of plastic additives that migrate to contacted food.^19,24−27^ However, there are very few reports of additive particle release in MP studies. Here, we hypothesize that MAPs released from plastics are potentially misassigned as MPs in MP studies due to the limitations of Raman spectroscopy. To test this hypothesis, we first characterized the Raman spectra of six typical additives and PE in bulk form and as micron-sized particles and found that their hit quality index values (0.93–0.96) are much higher than the accepted threshold value (0.70) used to identify MPs in microplastic studies. To prevent this interference, we introduced a new protocol that includes an alcohol rinse step that successfully separates interfering MAPs from the MPs and allows them to be separately analyzed. We then investigated three typical plastic products (i.e., PE pellets, PE caps, and polypropylene (PP) food containers) using the new protocol to confirm the simultaneous release of MAPs and MPs regardless of the plastic type, daily-use scenario, or service duration. This study not only reveals the hidden problem associated with the interference of additives in the detection of MPs but also provides a cost-effective method to prevent this problem.

## 2 Materials and Methods

### 2.1 Precautions for Contamination Prevention

During the experiments, the following steps were followed to avoid any potential MP contamination: boro 3.3 glassware was chosen for sample preparation; thoroughly cleaned particle-free nitrile gloves and cotton-based laboratory coats were worn during experiments. All water samples were covered with glass lids, and all filtered samples were stored in glass containers. A blank control sample was analyzed every 10 samples by filtering 100 mL of DI water. PE MPs were detected in blank control samples with an average level of 107 MPs per liter, while no PP MPs were found in control studies.

### 2.2 Studies on Standard MPs, MAPs, and Real-World Plastic Products

#### 2.2.1 Preparation of Standard MP and MAP Samples

To investigate standard PE MPs, 5 mg of additive-free standard PE spheres (Cospheric, a size range of 3–16 μm) were dispersed in 1 L of DI water (25 °C) and recaptured using a gold-coated polycarbonate (PC) membrane filter (APC, a pore size of 0.8 μm, herein referred to as the filter). The captured standard PE MPs were then characterized using multiple techniques, such as Raman spectroscopy and atomic force microscopy (AFM). Following the similar method, standard microsized particle samples of behenamide (Merck), stearamide (TCI), erucamide (TCI), oleamide (TCI), stearic acid (Merck), and erucic acid (TCI) were also prepared and investigated.

To validate the newly developed protocol, samples were prepared by mixing standard stock PE spheres and stearic acid additives in DI water and tested. The standard stock PE sphere solution was prepared by evenly dispersing around 10 mg of standard PE spheres (in powder form) in 1 L of DI water via sonication. The PE sphere concentration (around 3000 spheres/mL) in this stock solution was determined by membrane filtration and Raman determination using established protocols.^7,28,29^ Test water samples were then prepared by mixing 1 mL of stock PE sphere solution and 100 mg of the stearic acid additive in 1 L of DI water (containing around 3000 PE spheres and 100 mg of the stearic acid additive per liter). The water samples were shaken at a speed of 150 rpm for 2 h (25 °C) before filtration and the test.

#### 2.2.2 Study of Plastic Food Containers

PE and PP are the most widely used plastics, accounting for 33 and 21% of the global market share, respectively.^20^ In this study, PP-based food containers, PE-based bottle caps, and standard PE pellets were chosen to investigate the potential interference of chemical additives in the identification of MPs released from plastic products.

Brand-new PP-based food containers (purchased from local stores) were cleaned thoroughly after removing the packaging. Mimicking daily-use scenarios, the clean containers were filled with 500 mL of DI water and microwaved for 5 min. After that, the containers were covered with a lid and shaken at a speed of 150 rpm for 5 min to mimic the shaking actions during users’ holding, moving, and eating. After cooling down, the water samples were filtered using a filter. Raman determination was performed following a typical MP protocol^29−31^ to characterize and quantify the particles on the filter. The filter was then placed onto a glass holder and rinsed using ∼30 mL of ethanol (high-performance liquid chromatography (HPLC) grade, in a glass bottle, Fisher Chemical). After the rinse, the remaining particles on the filter were again characterized and quantified using Raman spectroscopy.

To determine the sources of MAPs, the container experiments were repeated 50 times following the same protocol detailed above. Detailed chemical analysis (before and after ethanol rinse) was undertaken on the water samples from the 10th and the 50th run.

#### 2.2.3 Study of Standard PE Pellets

For standard PE pellets (low density, Merck, nominal size of 5 mm), 5 g of pellets were placed in 80 mL of DI water in a glass bottle, which was then shaken at a speed of 150 rpm for 4 h (25 °C), consistent with previous reports.^32,33^ The pellets were removed from the water sample using a stainless steel mesh (mesh size of 1 mm). The particles released into the water samples were captured using the filter and characterized using multiple techniques. Similar to the container tests, the particles were analyzed before and after an ethanol rinse. Before filtration, the water samples were also tested using a total organic carbon (TOC) analyzer to determine the concentration of organic carbon (C) and nitrogen (N). In addition, the ethanol filtrate from the rinse was drop-cast on a gold-coated substrate, air dried, and tested using Fourier-transform infrared spectroscopy (FTIR).

#### 2.2.4 Study of Water Bottle Caps

Bottled water was purchased from local stores and the PE-based water bottle caps (*n* = 4) were carefully rinsed using DI water and placed in 100 mL of DI water in a glass flask. The flask was then shaken at a speed of 150 rpm for 4 h (25 °C). The particles released into the water samples were captured using a filter and characterized using multiple techniques. Similar to the container tests, the filter was investigated before and after an ethanol rinse. In addition, gas chromatography–mass spectrometry (GC–MS) tests were also conducted to determine the composition of alcohol-dissolved MAPs released from the PE cap samples.

### 2.3 Alcohol Treatment to Eliminate the Interference of MAPs on MP Detection

Two types of alcohol treatments were conducted: an alcohol rinse and in situ ethanol test. During an alcohol rinse, the filter containing captured particles was placed onto a glass holder and rinsed using ∼30 mL of alcohol (ethanol or methanol). In the case of in situ ethanol tests, a target particle or a specific region of the filter was exposed to 1 drop of ethanol (20 μL) and was immediately reimaged after air drying.

### 2.4 Characterization and Determination of MPs and MAPs

#### 2.4.1 Determination of MPs and MAPs Using Raman Spectroscopy in Our Lab

A calibrated Raman spectrometer (Renishaw inVia) equipped with a charge-coupled device (CCD), an upright microscope (NT-MDT), and a 532 nm laser (Coherent Inc.) was used to identify and quantify MPs captured on the filter surface. For typical particle detection, the accumulation was set to 3 times and exposure time set to 10 s, which is similar to the typical test setting for MP determination.^7,28,29^ For some particles with weak signals, the accumulation time was increased to obtain clear spectra. The spectra were measured in the range of 500–3500 cm^–1^. Referring to previous studies,^30,31,34^ the hit quality index (HQI) value of 0.70 was set as the threshold for identifying a particle as a potential MP. The HQI was obtained by conducting a Pearson correlation analysis (OriginPro 8.6) between the target particle and the standard polymer. If necessary, the Raman spectra background of target particles was subtracted before analysis (LabSpec 5). Given the high similarity between MAPs and MPs, in situ ethanol tests and manual spectra checks were further conducted to confirm whether particles were MPs. To identify an MP, around 20 μL of ethanol was drop-cast on the target particle. After air drying, if the morphology changed substantially, additional ethanol was added until there was no significant morphological change. Then, the particle’s Raman spectra were collected and compared with the known fingerprint spectra of standard parent polymers. For example, for potential PE MPs, characteristic peaks at around 1415, 1440, and 1460 cm^–1^ were checked, which are associated with the CH_2_ vibrations in PE and the level of PE polymer crystallinity.^35,36^ To quantify the MP levels, four representative spots (typically two spots in the middle area and two spots close to the edge of the filter with a total tested area of 1.5 mm^2^) were analyzed. After the Raman test, the total number of MPs in the tested area was obtained using software ImageJ (US National Institutes of Health). Finally, the MP level per liter was calculated based on the water sample volume, total area of the filter, tested filter area, and confirmed MP numbers. Following this protocol, the recovery rate of tests involving standard PE microplastic spheres can reach 93.8%, as previously detailed.^37,38^

#### 2.4.2 Determination of MPs and MAPs Using Other Technologies

A scanning electron microscope (SEM, Zeiss Ultra Plus), an FTIR instrument (PerkinElmer), TOC analyzer (Shimadzu, TOC-L), AFM (NT-MDT), and GC-MS (Shimadzu) were used to characterize and determine MPs and MAPs, and the test conditions are detailed in the Supporting Information.

## 3 Results

### 3.1 Challenge of Raman Spectroscopy to Distinguish MAPs and MPs

**Figure 1:**
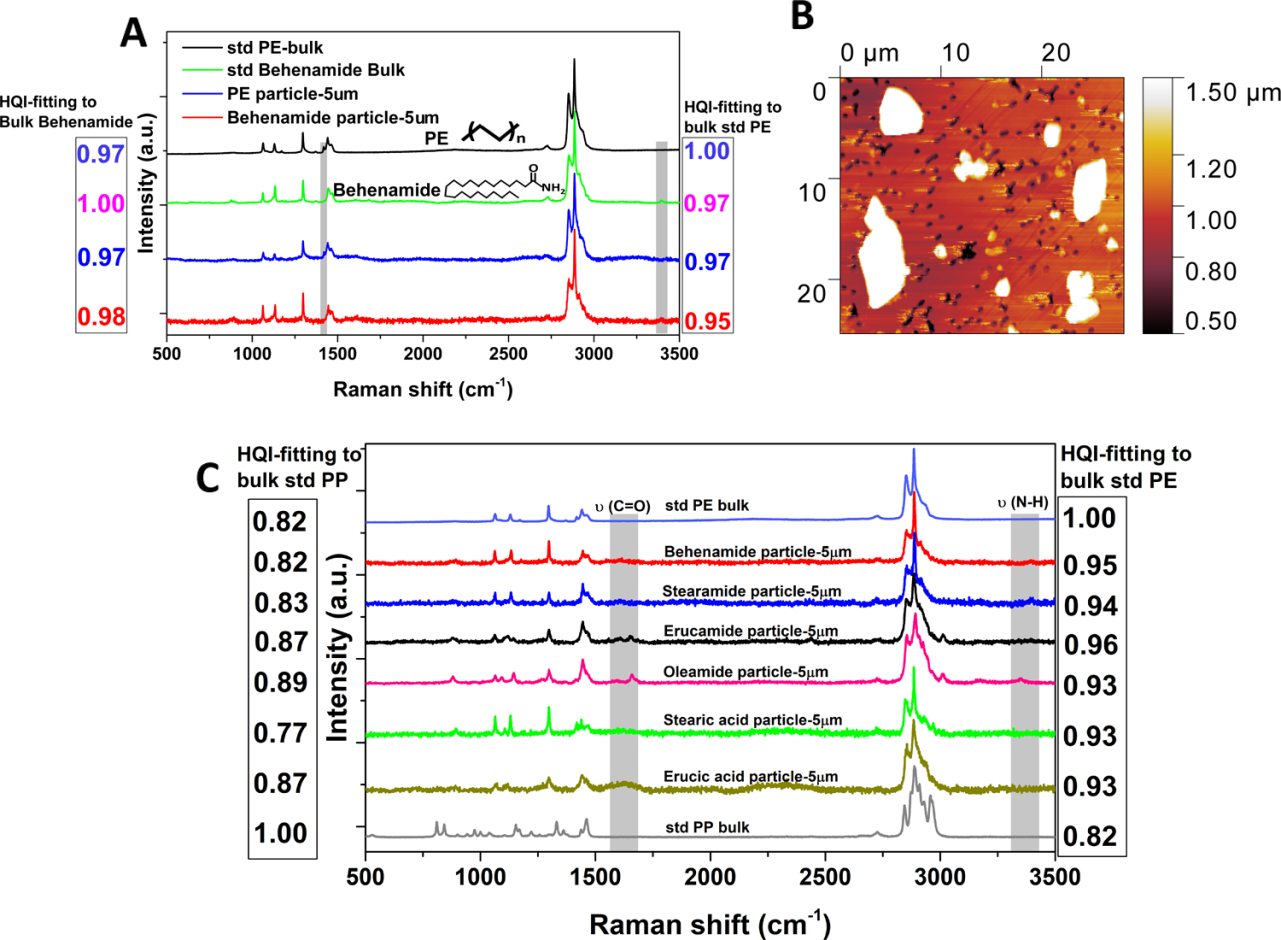
(A) Raman spectra of PE and behenamide in bulk and microsized particles. The standard bulk PE sheet was obtained from Goodfellow. (B) AFM image of behenamide microparticles on a filter surface. (C) Raman spectra of MAPs from six typical slip additives and standard PE and PP sheets.

We begin with a Raman analysis of standard PE and behenamide (one typical slip additive used in plastic, Figure 1A). The Raman spectra of these two bulk materials showed minor differences. PE showed a small peak at 1415 cm^–1^ associated with the vibration of CH_2_,^36,39^ while there is a minor peak in the behenamide spectrum at around 3415 cm^–1^ associated with the NH-amine vibration. However, even for the bulk materials, these differences are too small to distinguish behenamide from PE. The HQI between the bulk behenamide and PE is 0.97, much higher than the typically accepted threshold value (0.70).^30,31,34^

Behenamide powder and standard PE spheres were then dispersed in DI water and recaptured using a Au-coated filter. Interestingly, a high number of irregular-shaped microsized solid particles were obtained from the behenamide sample (Figure 1B), which are remarkably similar to the shape of MPs reported in previous publications.^40,41^ When the particle size decreases to the microscale, the two small Raman peaks differentiating the materials in the bulk spectra become even weaker and close to the signal-to-noise ratio level under a typical Raman test setting used in MP studies.^7,28^ For a 5 μm particle of behenamide, the HQI compared to the PE bulk can reach 0.95, as its spectrum is very similar to that of a standard PE sphere (HQI = 0.97, compared to the PE bulk). Evidently, it is very difficult to distinguish between behenamide MAPs and PE MPs on the basis of Raman spectroscopy.

In addition to the behenamide test, five typical slip additives were also separately dispersed in DI water, recaptured, and tested using Raman spectroscopy. In comparison to standard PE, the HQIs for all additive particles were in the 0.93–0.96 range (Figure 1C), much higher than the accepted threshold value (0.70). Moreover, the HQIs of these additives compared to standard PP are around 0.8–0.9, and so these particles may also be easily misassigned as PP MPs. This confirms that this interference problem is common across a wide range of chemical additives typically incorporated into plastics. Improvement in detection and analysis may be achieved by specifically analyzing narrow frequency ranges associated with characteristic peaks (e.g., 1000–1500 cm^–1^ to distinguish between PE and slip additives, Figure S1), enhancing the equipment sensitivity and modifying test protocols and analysis algorithms, but it is very difficult for Raman spectroscopy to distinguish between chemical additives and MPs based on the currently used MP test protocol.^29−31^

While most additives are insoluble in water (solubility <0.0005g/100g (0–60 °C) for stearic acid in water,^42^Figure S2), they can be dissolved and extracted using alcoholic solvents (solubility of 2.3g/100g (20 °C)–400g/100g (60 °C) for stearic acid in ethanol^42−45^). Tests using filter-captured oleamide particles show that they are dissolved and removed in a few minutes after exposure to just 1 drop (20 μL) of ethanol. Crucially, exposure to ethanol has no impact on MPs, as evidenced by the stability of the 5 μm standard PE sphere after repeated in situ exposure tests to ethanol (Figure S3). This is consistent with a previous report that most polymers are highly resistant to ethanol and methanol.^46^

### 3.2 MAP Release and Its Interference in the Detection of MPs from Plastic Products

**Figure 2:**
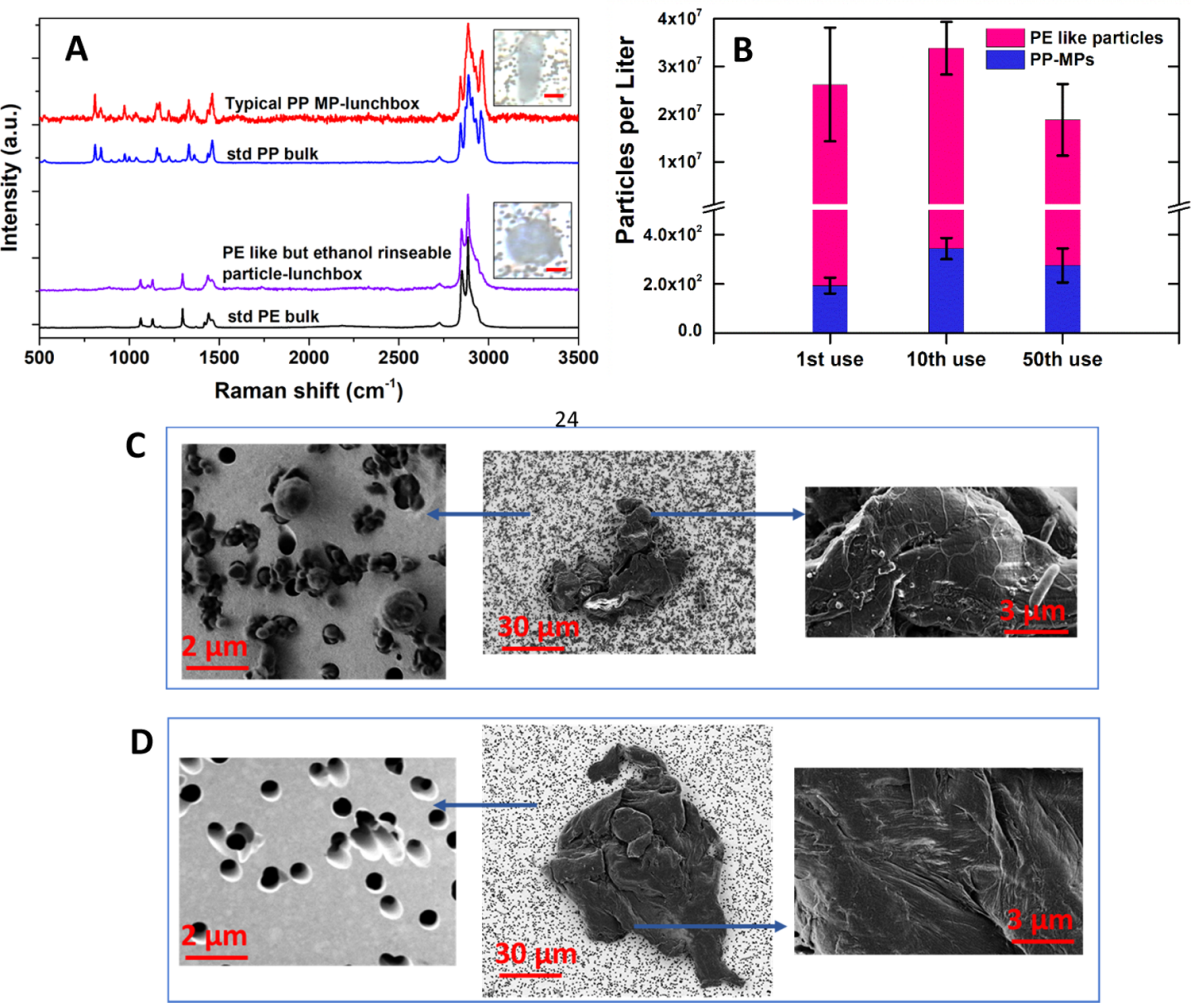
(A) Typical Raman spectra of PE-like particles (from plastic food containers), PP MPs (from plastic food containers), and standard PE and PP bulk sheets, respectively. The standard bulk PE and PP sheets were obtained from Goodfellow. The scale bars in the inserted images are 5 μm. (B) Quantities of PE-like particles and PP MPs during the 1st to the 50th test of the plastic food containers. (C) SEM images of particles released from standard PE pellets before (upper panel) and (D) after (lower panel) an ethanol rinse. All particles were on a Au-coated PC filter membrane.

PP-based food containers were investigated to check for MAP interference under real-world conditions. Raman-based chemical determination following the typical MP protocol^29−31^ confirmed high quantities (26,000,000 ± 11,900,000 per liter) of PE-like MPs released into the water samples during the first use. Compared to the standard PE, the HQIs of most tested particles were over 0.95 (Figure 2A,B). It is also noticeable that the HQIs of tested particles can reach 0.85–0.89 compared to standard PP, and so these particles could be misassigned as PP MPs since they originated from a PP-based container. This high MP release level is comparable to previous reports on plastic containers (releasing over 1 million MPs per liter^26,47^ or 1.2–7.6 mg of MPs from a single container^48^). However, most of these confirmed MPs dissolved away when the filter was placed onto a glass holder and rinsed using ethanol. FTIR tests of the dissolved particles showed a significant peak associated with the carbonyl (C=O) group (1740 cm^–1^), and library fitting indicates that they are slip additives such as stearic acid (Figure S4). After the ethanol rinse, a Raman analysis of the filter confirmed that the PE-like particles (HQIs > 0.95) were completely removed. The remaining particles (230 ± 30 PP MPs per liter) exhibited HQI values compared to standard PP that were higher than 0.95, exhibiting clear characteristic peaks at around 810 and 840 cm^–1^ associated with CH_2_ rocking, C–C stretching, and crystallinity of PP.^49^ Evidently, MAPs can substantially interfere with the determination of MP levels, and in this case up to 5 orders of magnitude difference in levels.

To check whether this interference is a short-lived effect due to the initial conditions of the manufactured product and/or the facile depletion of MAPs from the product surface region, the food containers were repeatedly used 50 times and the water samples from the 10th and 50th runs were chemically analyzed. Without the ethanol rinse, the PE-like MPs were always higher than 18 million per liter during the 50 runs. This result contrasts strongly with the low MP release levels after the ethanol rinse, with only 390 ± 40 and 340 ± 70 of PP MPs released during the 10th and 50th use, respectively. On this basis, there is a consistent level of MAPs released and the interference phenomenon persists over time. This is consistent with the ability of slip additives to continuously migrate to the surface, even though they are generally blended inside the bulk polymer.^50^

Distinguishing between PE-like MAPs and PE MPs released from PE parent plastics is even more difficult by Raman spectroscopy. Due to their widespread use in MP studies,^32,33,51,52^ commercial standard PE pellets were investigated by shaking in DI water (25 °C). Two types of particles were released: small ball-like particles (1–3 μm) and large irregular fragments (50–100 μm) (Figure 2C). Raman determination confirmed that both types of particles are PE MPs, with HQIs compared to standard PE for the small ball-like particles and large fragments of 0.89 and 0.88, respectively (Figure S5). However, most of the small ball-like particles were ethanol soluble. FTIR filtrate tests showed significant peaks associated with carbonyl (C=O) (1750 cm^–1^) and amide (N–H_2_) groups (3200–3500 cm^–1^), while a total organic carbon–total nitrogen (TOC–TN) analyzer confirmed that the carbon to nitrogen molar ratio (C/N) of these ball-like particles is around 22:1. These results indicate that they are likely behenamide or erucamide. As to the large irregular fragments, the quantity and size distribution remained unchanged after the ethanol rinse. Moreover, Raman spectra of the insoluble large fragments exhibited an HQI of around 0.9 compared to standard PE, which indicates that these fragments are real PE MPs.

Raman-based tests of water bottle caps (a typical PE product) also confirmed that high quantities of PE-like particles are released in the water samples. These PE-like particles were easily dissolved by alcohols, which confirmed that they are MAPs. GC–MS tests showed that the retention time of the main peak from the MAPs released from cap samples was 20.5 min (Figure S6). Mass-to-charge ratio (*m*/*z*) analysis shows that the chemical composition of the main peak matched that of oleamide (Figure S6, with a similarity of 94/100). Evidently, the release of MAPs is a pervasive phenomenon regardless of plastic types, daily-use scenarios, and service duration.

### 3.3 MAPs Modify the Surface of MPs

**Figure 3:**
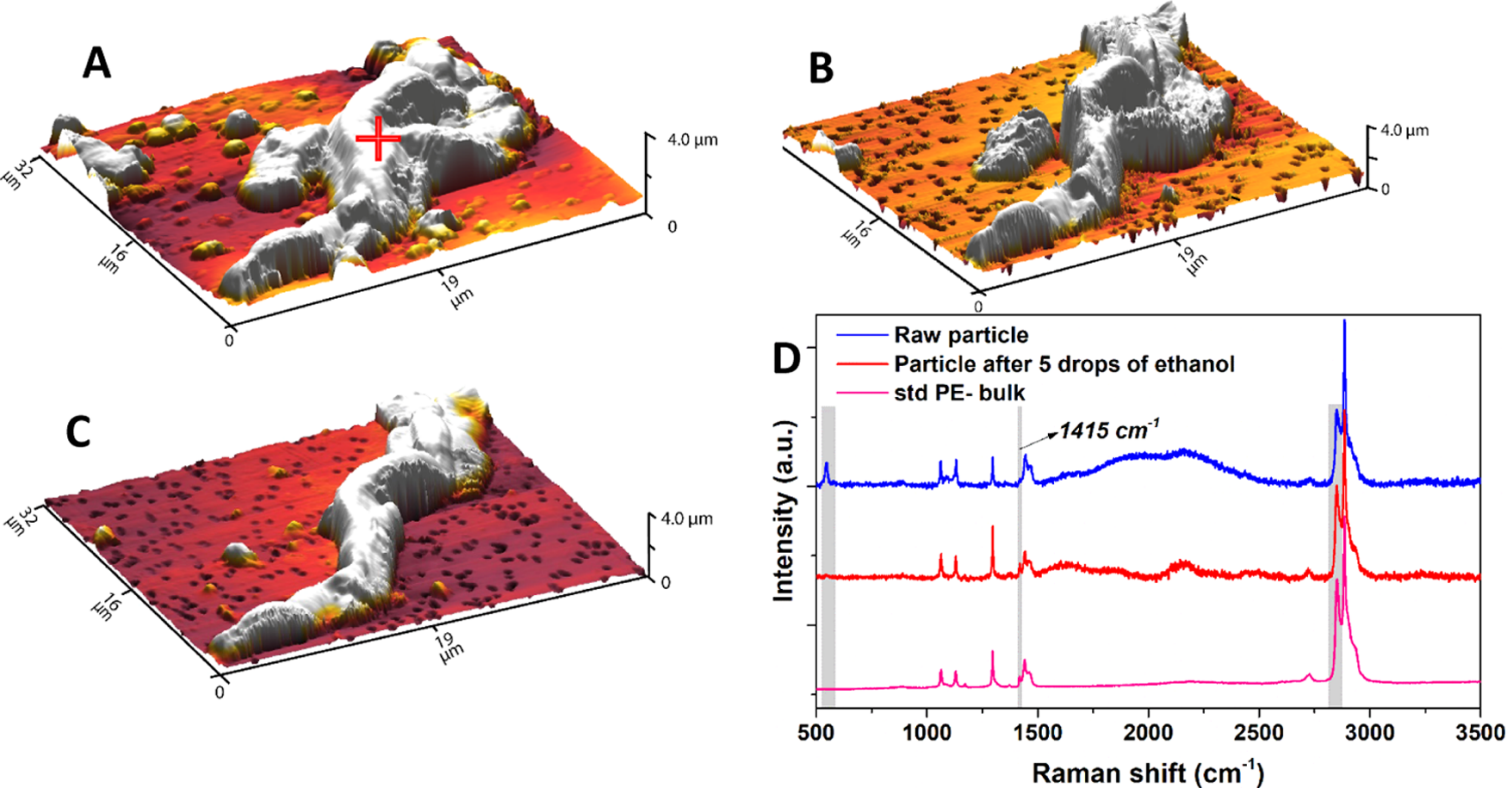
In situ test of microplastic and additive mixture particles released from the water bottle cap. (A) Raw particles captured using a Au-coated PC filter. (B) Particle changes after the drop and drying of 1 drop of ethanol. (C) Particle changes after the drop and drying of 5 drops of ethanol. (D) Raman spectra of the raw particle, particle after 5 drops of ethanol, and standard PE, respectively.

MAPs can attach to and modify the surfaces of MPs. Figure 3 shows a large irregular-shaped particle (labeled + in Figure 3A) released from a water bottle cap. The Raman spectrum from the labeled particle is similar to that of the parent polymer (PE). However, the peak at 1415 cm^–1^ associated with the vibration of CH_2_ from PE is very weak, which indicates that this particle may be an MAP or MAP/MP mixture. After exposure to 1 drop of ethanol (Figure 3B, an in situ test), the particle topography was substantially changed, with the reduction on the right side of the particle and the emergence of a separate smaller particle on the left. Following additional ethanol treatment, the big irregular fragment was transformed into a fiberlike shape, with the loss of material on the left and right (Figure 3C). After 5 drops of ethanol, further ethanol treatment resulted in no significant change in the fiber shape. Raman analysis of the ethanol-exposed particle showed that the PE vibration of CH_2_ at 1415 cm^–1^ is visible, which indicates that the mixed additive coating has been removed from the surface of the PE particle. Similarly, in the case of PE pellet samples, it is evident that smaller particles can attach to the surface of large MPs (Figure 2C,D). After the ethanol rinse, the attached particles are completely removed, which indicates that they are likely MAPs. Organic surface coatings on MPs can substantially enhance the cellular internalization capacity of MPs.^53,54^ Recent studies have also shown that the MP shape is a critical determinant of health risks in humans.^55^ Evidently, alcohol pretreatment can reveal/remove MAPs attached to MP surfaces, which enables the real morphology and risks of MPs to be determined.

### 3.4 Protocol to Test MAPs and MPs Separately

**Figure 4:**
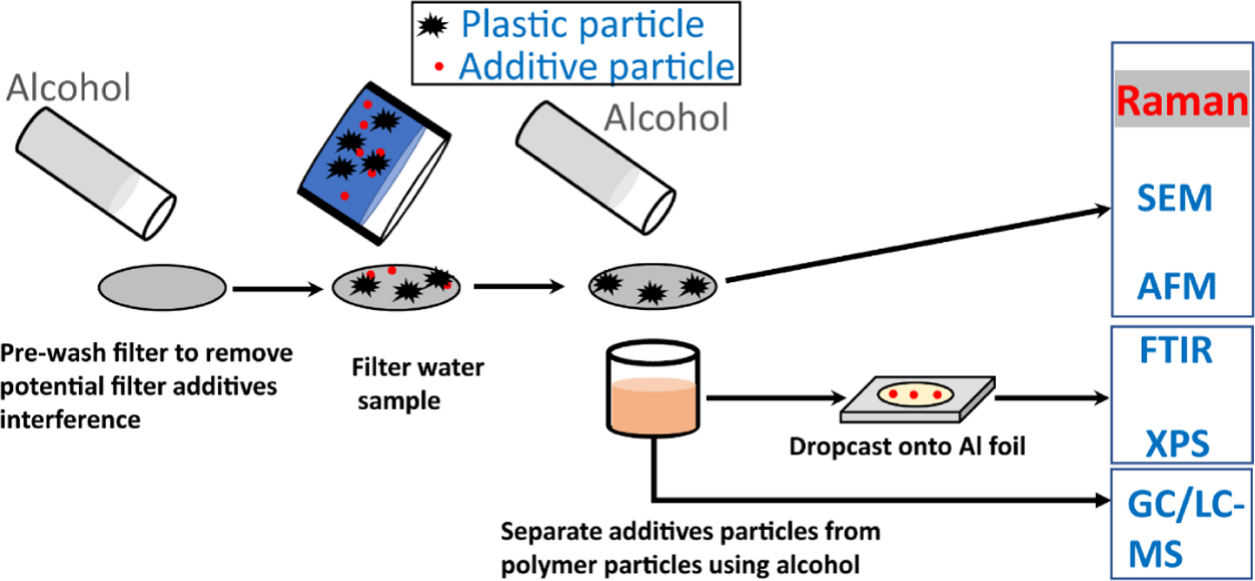
Protocol to separate additive particles and plastic particles.

To effectively separate MAPs and MPs and prevent misassignment, an alcohol rinse step was incorporated into a modified protocol (Figure 4). The filter was first washed using an alcohol solvent to remove any possible additive contamination in the filter. After that, the filter was used to capture the MPs and MAPs from the water sample. The captured particles were then rinsed with alcohol. Tests using real samples (food containers, PE pellet samples, etc.) confirmed that a rinse with ∼30 mL of ethanol/methanol for 30 min is sufficient to remove the interfering MAPs. Finally, the residual particles on the filter surface were analyzed using Raman spectroscopy while the alcohol filtrate containing the dissolved MAPs was analyzed using other techniques (i.e., GC–MS).

To validate this protocol, a water sample was prepared by mixing standard PE spheres (3–16 μm) and stearic acid in DI water. The PE sphere level was 3000 particles per liter, which is similar to the MP level previously reported.^56^ Following the proposed protocol, the PE spheres were collected and counted yielding a recovery rate of 93.8%, which is comparable to the previous report.^57^ In addition, the presence of stearic acid dissolved in the methanol rinse was also successfully identified using GC–MS (Figure S7). As to the topography of PE spheres, the original round and smooth surfaces were initially modified by MAP attachment but fully recovered to the original smooth shape after the methanol rinse (Figure S8). Hence, the developed protocol can successfully and effectively separate additives from MPs and avoid misassignment, while facilitating the characterization of the true topography of MPs.

## 4 Discussion

### 4.1 Challenges to Avoid MAPs in MP Detection

Accurately mimicking real-world conditions is critical to quantitatively determine the levels of MPs released from plastics, including their true morphology. For example, in the manufacturers’ instructions for microwaveable PP food containers, the contents (water and/or food) are suggested to be thoroughly heated and allowed to cool down before consumption. During this cooling down period, the plastic container will continue to release MPs and MAPs at a rate and level following which an accurate assessment of the risk to the consumer is possible, including the formation of MAP/MP composite particles where MAPs become attached to the surfaces of MPs (Figure 3). This is crucial to accurately assess MP translocation inside the human body and for a rigorous risk assessment of MPs to human health.^53^

In this paper, we identified that plastics can release up to 5 orders of magnitude higher levels of MAPs than MPs (Figure 2). Moreover, the small quantities of released MPs may be blended or even covered by MAPs (Figure 3), making it extremely difficult or even impossible to find and cleanly detect MPs using Raman spectroscopy. There are two possible contributions to this behavior: MAPs are initially released at the surface as mobile molecular species or clusters that aggregate into very small particles (less than 20 μm) when they come into contact with water (Figure 2). Another possible contribution is the migration of additives directly from the bulk to creep/cover the plastic surface.^22,25,50,58^ Our studies showed that an initial prewash of the plastic part/product in ethanol had no substantial effect on the subsequent MAP release, demonstrating that there is facile transport to the surface, consistent with the dominant release of MAPs over MPs. Longitudinal studies will be required to determine the exact profile of the MAP release and whether the level wanes after extended use, beyond the 50 product cycles reported in this work (Figure 2B)

Although typical slip additives were characterized here, the potential interference from other additives is also expected given that over 400 additives are used in just 10 types of plastics.^59^ For example, BHT is a typical antioxidant widely used in PP. The HQI of BHT to PP is around 0.8, higher than the accepted threshold value. This indicates that water-insoluble BHT could potentially interfere in the determination of PP MPs.

Great care must also be taken in the analysis of environmental and other organic-rich samples such as the ground/marine water, sediment, and soil samples. Particle separation based on density differences is widely used in the MP study.^60^ However, this method is not able to separate MAPs from MPs efficiently as these MAPs have a similar density to low-density polyethylene (LDPE) (Table S1). In addition, the melting points of behenamide and stearamide are similar to PE, while others are lower than that of PE. This indicates that the temperature-based separation method may only be useful for certain additives. As for digestion, most studies (∼60%) did not involve a digestion process to remove the organic material during sample isolation and preparation,^6^ which means that there is potential blending and interference of MAPs during MP detection. Only a small portion of these studies involve a sample digestion process aimed at removing environmentally introduced organic matter but even this will likely fail to remove MAPs effectively. For example, digestion of behenamide using three typical chemicals (H_2_O_2_, HCl, and NaOH, 1 M, 60 °C, 24 h, following typical digestion processes^57,61−63^) fails to remove behenamide MAPs effectively.

In addition to microsized particles, the interference of nanosized MAPs (NMAPs, <1000 nm) is also likely. Raman spectroscopy is the most widely used nondestructive technique for NMP characterization.^8−11^ Our case study confirmed that the high quantities of NMAPs are released from PE plastic (Figure S9). However, the potential interference of NMAPs in NMP determination is expected to be even greater given that the signal/noise ratio decreases as the MP size decreases (Figure 1). Hence, the prevention of NMAP interference is also essential.

### 4.2 Misassignment of MPs and MAPs

Given the similarity between some MAPs and MPs, the high quantity of MAP release, and the lack of preventive steps (∼60% of MP studies did not involve a digestion pretreatment^6^), it is likely that MAP interference played a significant role in many previous MP studies. For some reported PE MPs confirmed by Raman analysis, there was no vibration of CH_2_ at around 1415 cm^–1^^7,9,10,28^ but instead, the presence of an extra peak at around 3100 cm^–1^.^7,28^ In addition, a peak around 1750 cm^–1^ associated with the carbonyl group was also observed in the case of other confirmed PE MPs.^28^ These minor changes in the Raman spectra are generally attributed to environmental weathering and/or the inclusion of additional compounds and pigments.^28^ However, it is evident that pure MAPs can account for these Raman features (Figures 1 and 2). HQI analysis based on a full spectrum comparison is widely used in MP studies but it is difficult to distinguish and account for minor differences (Figures 1–3). Additionally, in some instances, just a few characteristic vibrational bands associated with the specific polymer were used for MP determination and chemical spectral mapping.^64−66^ However, comparing MAPs of typical slip additives with standard PP using the spectral range between 2500 and 3200 cm^–1^ (Figures 1 and 2), the characteristic peaks at 2720, 2850, and 2880 cm^–1^ perfectly match that of PP. The comparison to standard PP using this narrow spectral range yields HQIs for typical slip additive particles (i.e., oleamide and erucic acid) of over 0.9, much higher than the accepted threshold value.

The MAP misassignment clearly occurred in studies of plastic food containers. There were over a million MPs per liter released in the present study and in previous reports.^26,47,48^ However, after an ethanol rinse, the MP level dropped by 5 orders of magnitude in this study. Similarly, while large quantities of MP were reported to be released from plastic baby feeding bottles,^3,37^ our reassessment based on the new alcohol protocol showed that the levels of MPs decreased from over 1 million per liter to around 100–100,000 per liter, which depends on multiple factors, such as the brand type and duration of use. Beyond the cases mentioned above, the problem of unintentional misassignment of MAPs in previous MP studies is significant and primarily due to the unanticipated limitations of the detection technologies and sampling protocols used. Evidently, a reassessment of the MP release levels is required, especially in instances where extremely high levels were reported.

The misassignment of MPs and MAPs can also result in an inaccurate risk assessment and false management given their different physicochemical properties and toxicity. First, while MAPs are particles, they are easily broken down into their constituent molecules. MPs are highly recalcitrant macromolecules that are inert, with very poor biodegradability.^67^ For instance, there was only negligible weight loss observed when PE was kept in moist soil for 12–32 years.^67^ In contrast, many MAPs have good biodegradability (European Chemicals Agency). For instance, over 70% of stearic acid mixed in soils can be biodegraded to CO_2_ within 3 months.^68^ The toxicity of MPs and MAPs to human health can be substantially different. For MAPs, it was reported that BHT chronically damages the liver, such as increasing the liver weight and enzyme activity.^17,69^ The chronic exposure to erucic acid can lead to myocardial lipidosis, though this effect is reversible and transient.^70^ As for erucamide, oleamide, behenamide, stearamide, and stearic acid, the EU Commission Regulation^18^ pointed out that they are allowed in the production of food-grade plastic materials, which indicates that they should have insignificant risk to humans. An assessment conducted by the Canadian Environment and Health Department also confirmed that erucamide and oleamide have low toxicity to human health.^71^ As for MPs, a previous study found that MPs smaller than 10 μm can translocate and accumulate in tissues, such as the liver and kidneys.^5^ However, the specific risks of MPs to human health are still unknown^5^ although the negative health impact of MPs on animals is widely reported.^72−74^ Currently, studies focusing on MP toxicity characterization use both commercial MP spheres and real irregular MPs generated from bulk plastics. However, there was no consideration given to the potential levels of additives in these MP samples. The types and concentrations of additives in MPs can be significantly different. For instance, antioxidant additives can range from 0.05 to 3% (by mass) in the same type of plastics.^20^ It is interesting to note that toxicity studies using the same type of MPs draw contradicting conclusions,^55^ which may reflect different levels of additives incorporated in plastics sourced from different manufacturers. Obviously, this requires further investigation. However, it should be noted that the mixing of MAPs and MPs and the coating of the latter by the former may enhance the cellular internalization capacity and alter the toxicity of MPs. Clearly, it is crucial to have an effective method to separate and investigate MPs and MAPs to accurately assess such risks.

### 4.3 Cost-Effective Method to Distinguish between MAPs and MPs

To date, there is still no consensus on a standard MP test method.^75^ Researchers and regulators are making huge efforts to develop reliable MP detection methodologies. Recently, the California Water Boards developed an MP analytical method that involves spiking water samples with typical polymer particles (PE, PS, etc.) and conducting a cross-laboratory validation.^76^ However, due to the omission of MAPs, this approach will likely be incapable of accurately determining MPs in real-world water samples that will unavoidably contain significant quantities of polymerlike MAPs.

Our proposed protocol with an alcoholic solvent rinse step achieved effective separation of interfering MAPs from MPs and enabled quantitative chemical analysis of each separately. Interestingly, 50% ethanol treatment was previously employed in an MP study to destroy the foam generated during filtration,^56^ which indicates that ethanol treatment may have multiple advantages in MP detection. While here we used a Au-coated PC filter, the alcohol rinse is also suitable for other types of filters, such as the aluminum oxide filter in our preliminary test. However, alcoholic solvents may not be effective for all MAPs used in plastics or other organic matter introduced from the surrounding environment. Further improvements are possible and achievable. Increasing the ethanol temperature is an effective method to substantially increase the solubility of many organic additives^42,45^ (Figure S2). It should be noted that directly adding ethanol into water samples at room temperature is not effective for removing MAPs due to the extremely low solubility of additives in the water/ethanol mixture regardless of the mixing ratio.^45^ However, increasing the temperature or changing the solvent can effectively increase the solubility. In addition, directly adding isopropanol into water samples at room temperature in the ratio of 50:50 (volume) should be feasible to remove MAP interference.^77^ Other solvents such as acetone and polysorbate 80 are also potential choices. While there are numerous solvent combinations, it is critical to ensure that the solvents do not damage the target MPs.

Taken together, we have demonstrated that additives and polymers, the two major components of plastics, can simultaneously release micron-sized particles, which substantially interfere with an accurate determination of MP release levels using Raman spectroscopy. Alcoholic solvent pretreatment is proved to be a cost-effective method to enable the separation, detection, and analysis of MPs and MAPs. By tuning the solvent types, pretreatment conditions, and filtration methods, researchers can systematically explore the release quantity/size, the attachment/detachment process, and morphological changes of MAPs and MPs under different conditions. Crucially, the approach provided here can be used to reassess the accuracy of previous MP studies and avoid potentially misleading results in future MP research.

## Supporting Information Available

Methods for in situ alcohol treatment; methods for particle characterization using SEM, AFM, FTIR, etc.; Raman spectroscopy, FTIR, and GC/MS test results for additives and microplastics, respectively; stearic acid solubility in water and ethanol; in situ ethanol test of standard PE spheres; SEM images of PE coated with additives and rinsed with ethanol; nanosized additive particles; and information summary of typical additives and polymers (PDF)
