# Supplementary material for: Alcohol Pretreatment to Eliminate the Interference of Micro Additive Particles in the Identification of Microplastics Using Raman Spectroscopy
Source: Environ Sci Technol. 2022 Aug 25;56(17):12158–68. doi: 10.1021/acs.est.2c01551 (PMC9454250; doi:10.1021/acs.est.2c01551)
Supplement: Supplementary file 1 — es2c01551_si_001.pdf [file es2c01551_si_001.pdf]

Supporting Information for

Alcohol pre-treatment to eliminate the interference of micro additive particles in the identification of microplastics using Raman spectroscopy

Dunzhu Li[1,2], Emmet D. Sheerin[1,4], Yunhong Shi[2,1], Liwen Xiao [2,3], Luming Yang[2,1], John J. Boland[1,4]*, Jing Jing Wang[1]**

[1]AMBER Research Centre and Centre for Research on Adaptive Nanostructures and Nanodevices (CRANN), Trinity College Dublin, Dublin 2, Dublin D02PN40, Ireland.

[2]Department of Civil, Structural and Environmental Engineering, Trinity College Dublin, Dublin 2, Dublin D02PN40, Ireland.

[3]TrinityHaus, Trinity College Dublin, Dublin 2, Dublin D02PN40, Ireland.

[4]School of Chemistry, Trinity College Dublin, Dublin 2, Dublin D02PN40, Ireland.

*Correspondence to: Jing Jing Wang, jjwang@tcd.ie; Liwen Xiao, liwen.xiao@tcd.ie; John J. Boland, jboland@tcd.ie

Number of pages: 12

Number of figures: 9

Number of tables: 1

Supporting Materials and Methods

In-situ alcohol treatment

In the case of in-situ ethanol tests, a target particle or a specific region of the filter was exposed to 1 drop of ethanol (20 μL) and was immediately re-imaged after air-drying. Most small molecular additives can be dissolved by ethanol [1] while typical polymers are very resistant to alcohol. In addition, the densities of most polymers are higher than that of ethanol so that the MPs remain in place on the filter. In contrast, the high solubility of MAPs means that alcohol can be used to selectively remove MAPs from an MP-MAP mixture (Fig. S2). For in-situ (single drop) tests, when the alcohol evaporates most of the MAPs are found to redeposit at the edge of drops due to the coffee-ring effect. Hence, the in-situ ethanol test was conducted as a quick check to determine whether there were MAPs present in the tested particles or filter area.

MPs and MAPs determination using other technologies

In addition to Raman spectroscopy, scanning electron microscope (SEM, Zeiss Ultra Plus) was performed with an acceleration voltage of 15 kV. FTIR instrument with a diamond in the attenuated total reflection (ATR, PerkinElmer) mode was used as a complementary spectroscopy to further confirm the chemical property of these MAPs. To study the concentration of total organic carbon (TOC) and the ratio of C/N, a TOC analyser (Shimadzu, TOC-L) was used to test the TOC and total nitrogen concentration in water samples. The morphology of released MPs and MAPs was acquired by atomic force microscopy (AFM, NT-MDT) with a tapping mode probe (Nanosensors, PPP-NCST). Gwyddion 2.54 software was used to analyse AFM results. GC-MS (Shimadzu) was conducted to determine the chemical identity of MAPs released from bottle water caps. Referring to a previous report [2], methanol

Figures

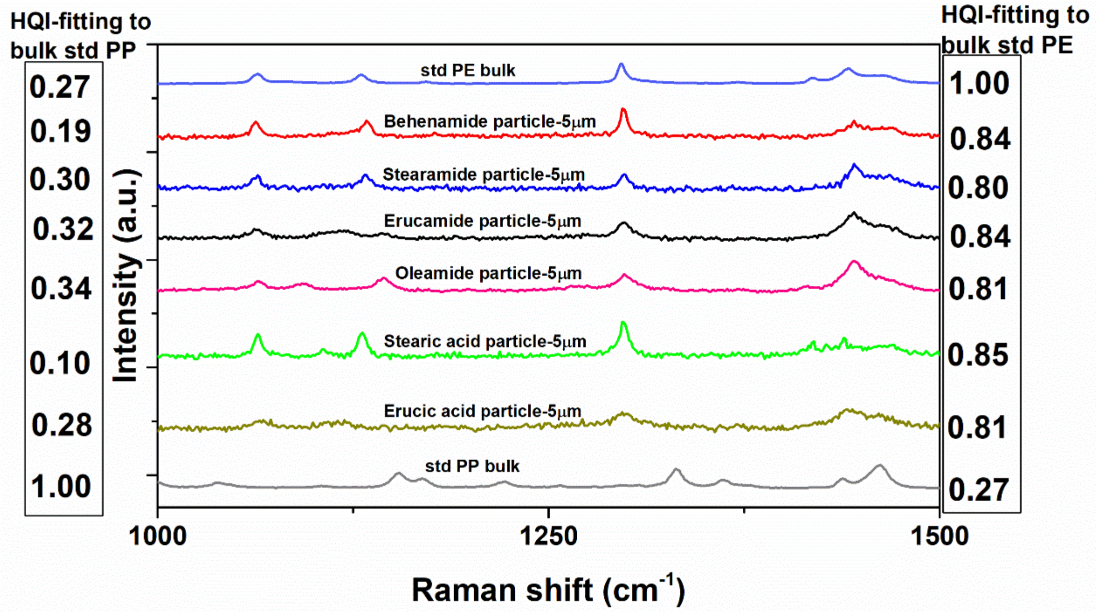

Figure S1. The narrow frequency range Raman spectra of MAPs from six typical slip additives and standard PE and PP sheet. Evidently, HQIs at the narrow range can be used to distinguish between PP and additives due to the values decreased to the range of 0.1-0.3, much lower than accepted threshold value (0.7). As to the comparison between PE and MAPs, though HQIs at the narrow range decreased slightly, it is still difficult to differentiate MAPs from PE because they are still much higher than 0.7.

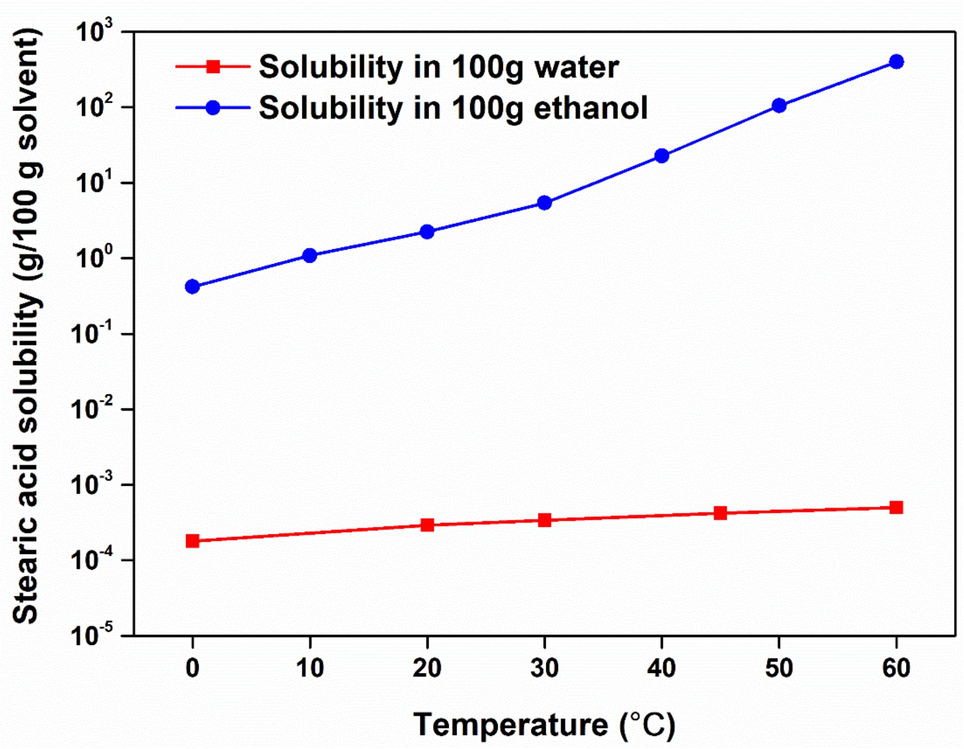

Figure S2. Stearic acid solubility in water and ethanol. Data extracted from [3-6] .

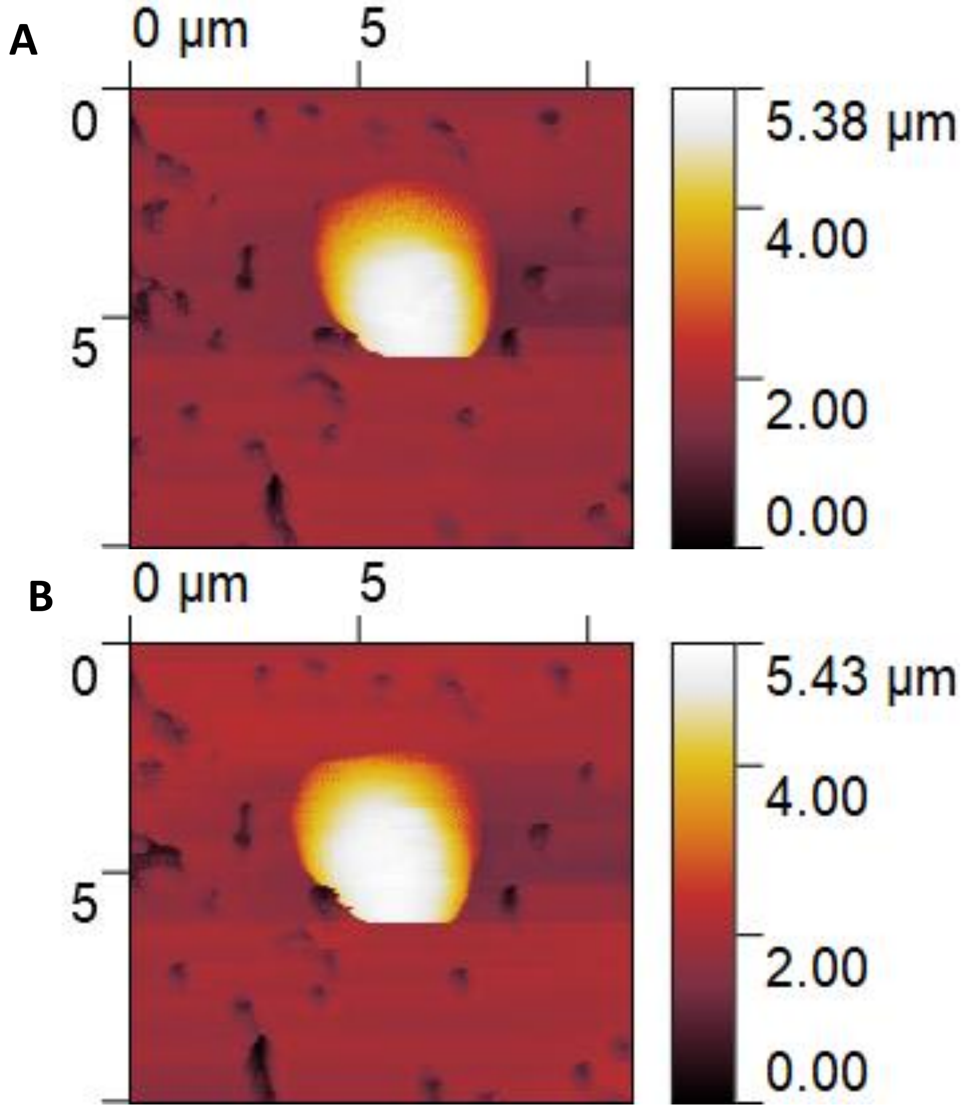

Figure S3. In-situ ethanol test of standard PE sphere. (A) Raw PE sphere captured using Au-coated PC filter. (B) Particle changes after the drop and dry of 20 drops of ethanol. Evidently, ethanol shows nearly zero change on the morphology and location of PE sphere.

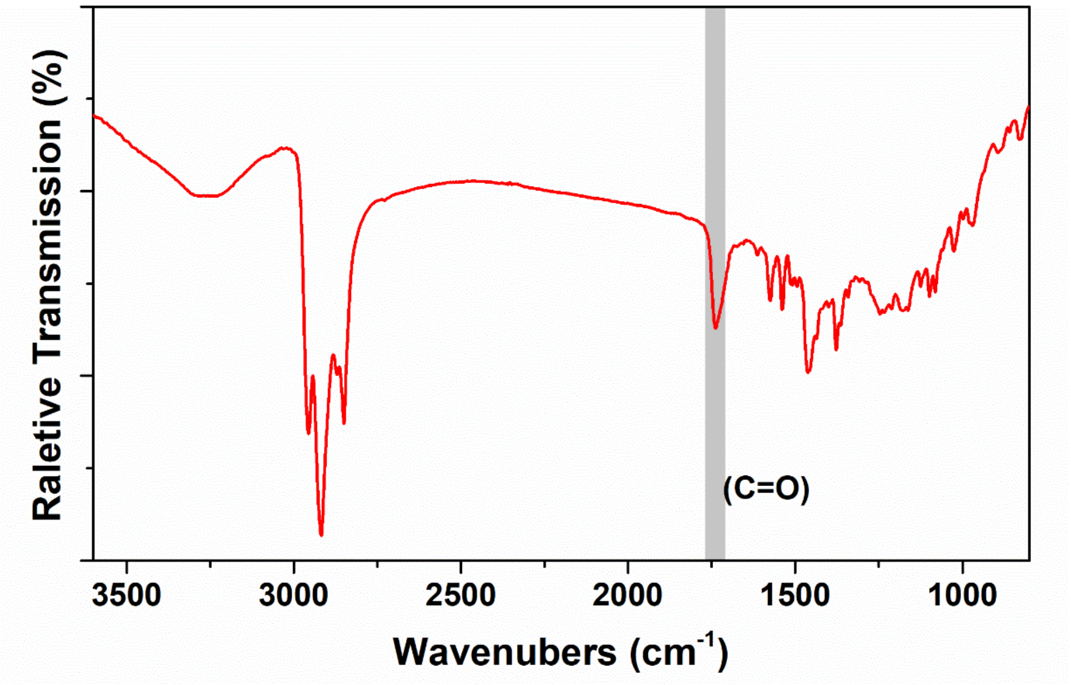

Figure S4. FTIR spectrum of the ethanol-rinseable particles from plastic food container.

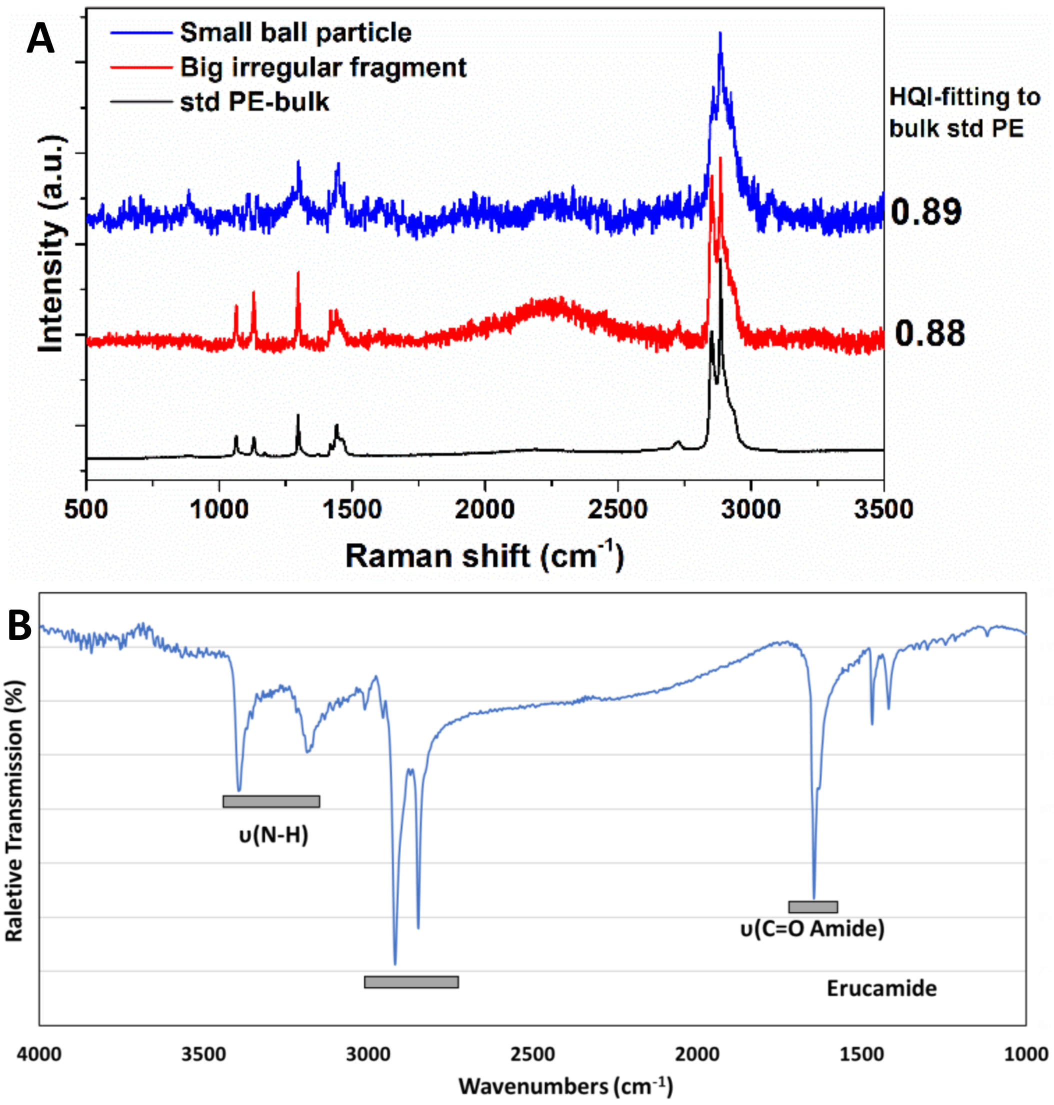

Figure S5. (A) Raman spectra of small ball particle and big fragment in Fig. 2c, respectively. (B) FTIR spectrum of the ethanol-rinseable particles.

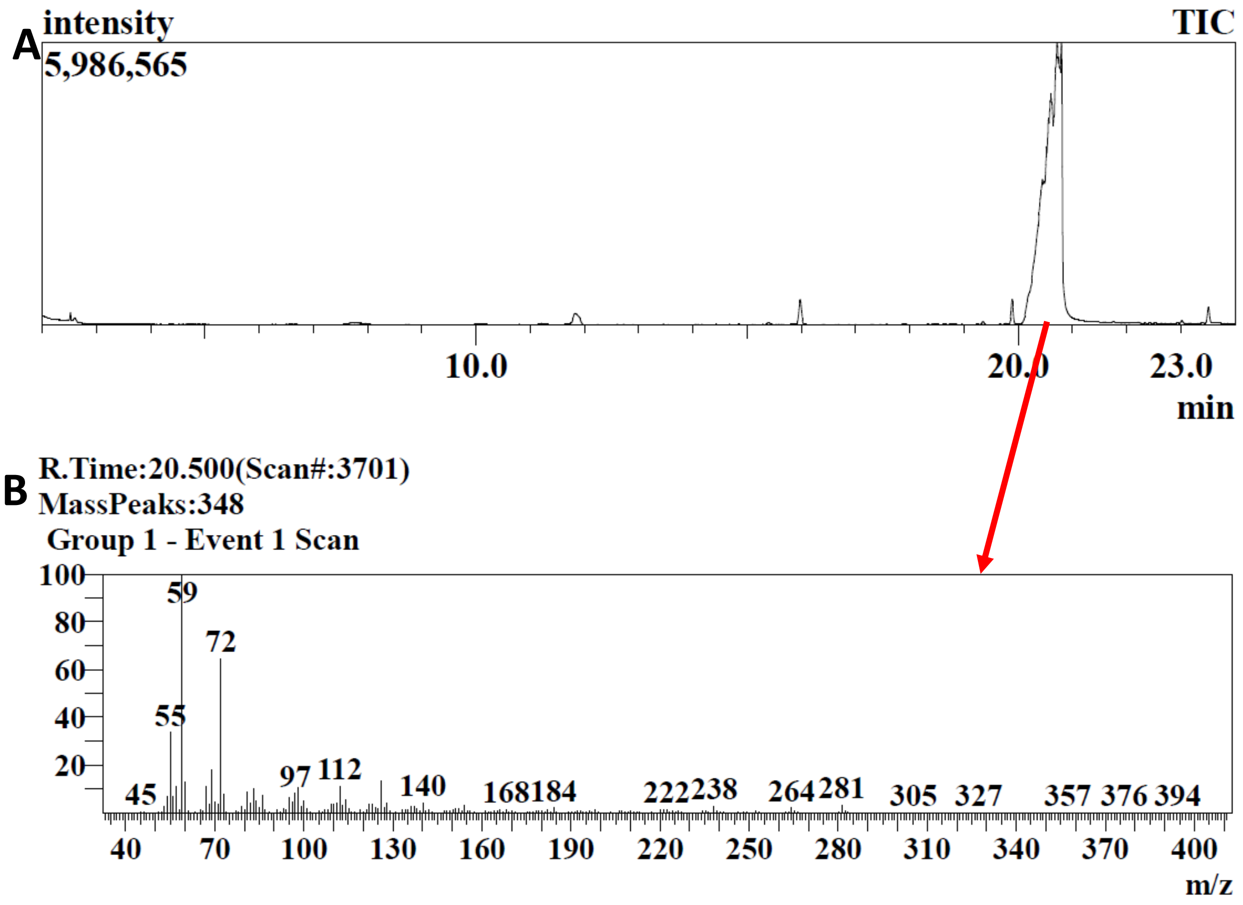

Figure S6. Typical gas chromatography/mass spectrometry (GC/MS) chromatograms from the bottle water caps water sample. (A) Gas chromatography profile of the methanol extracted MAPs. (B) Mass spectrogram at the peak with retention time of 22.50 min was selected and chemical compounds were determined by matching the mass spectra to the database of National Institute of Standards and Technology library.

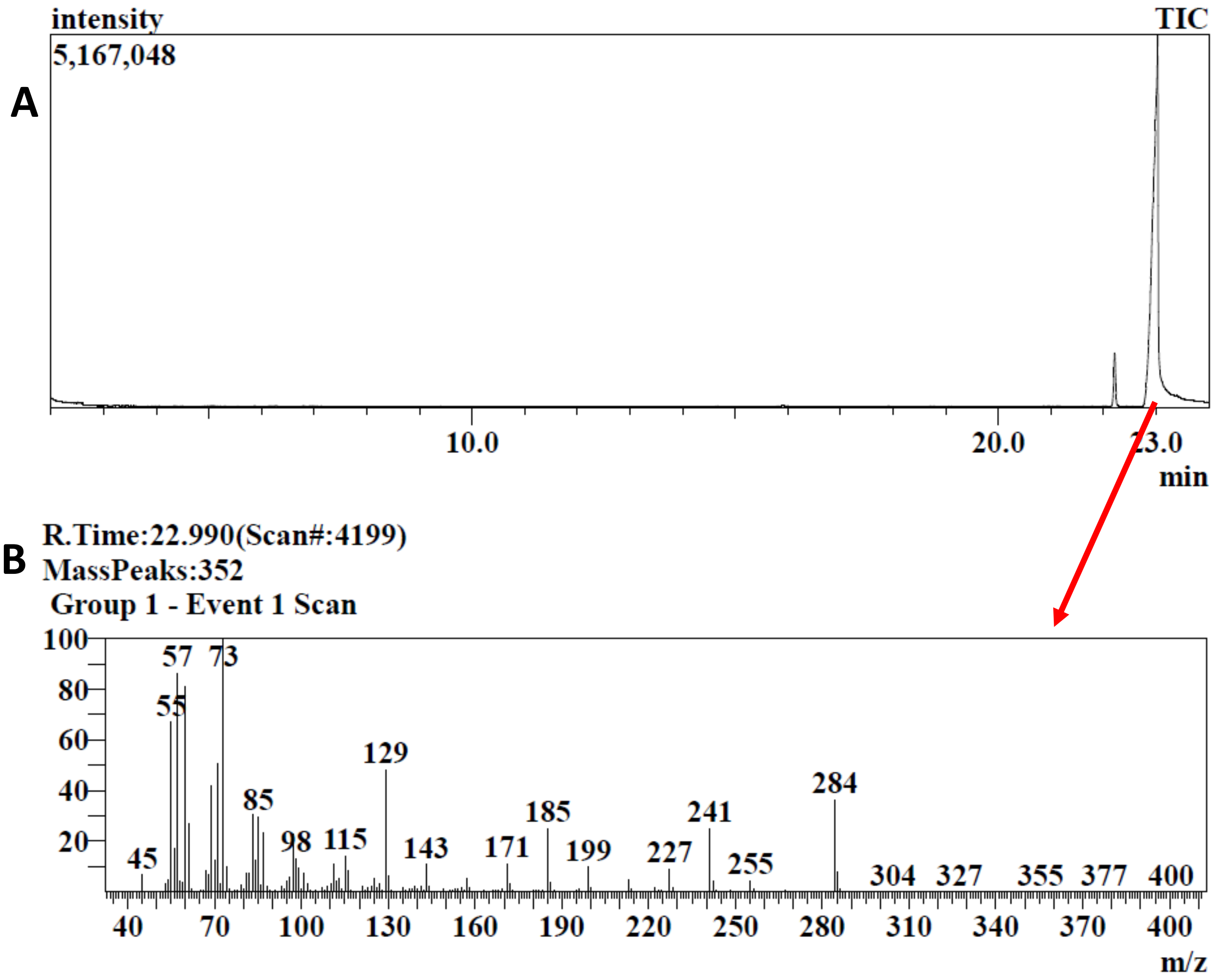

Figure S7. Typical gas chromatography/mass spectrometry (GC/MS) chromatograms from the simulated water sample containing standard stearic acid and PE sphere. (A) Gas chromatography profile of the methanol extracted MAPs. (B) Mass spectrogram at the peak with retention time of 22.99 min was selected and chemical compounds were determined by matching the mass spectra to the database of National Institute of Standards and Technology library.

A

B

C

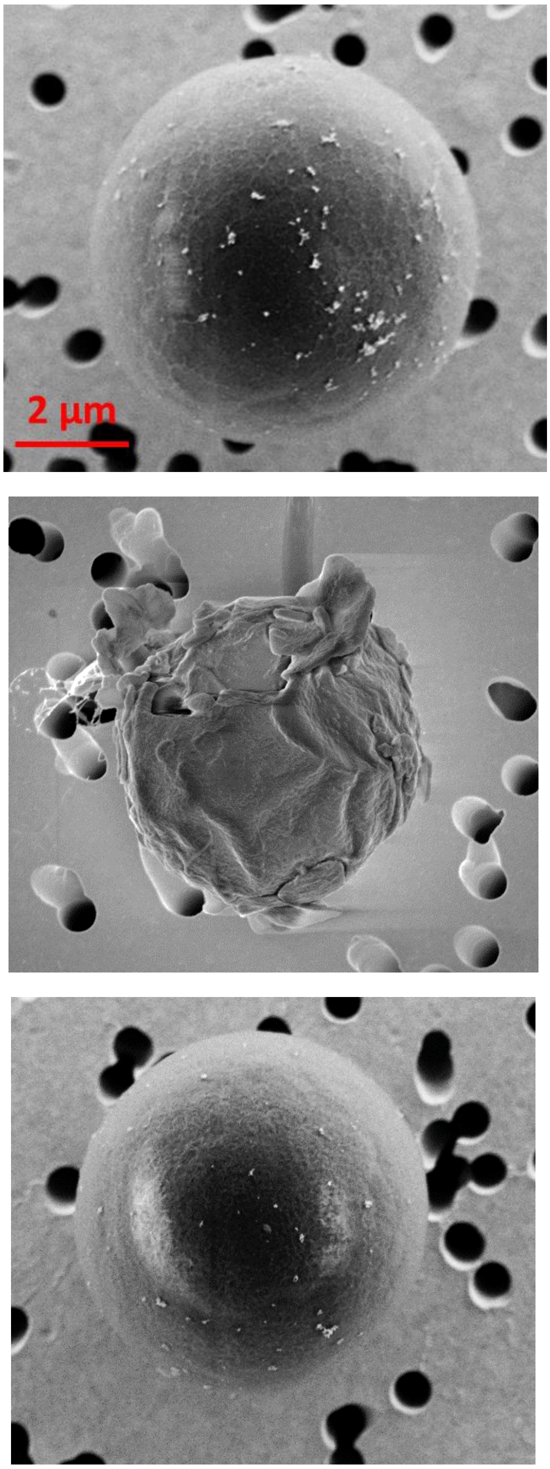

Figure S8. (A) Standard PE sphere (additive-free). (B) Standard PE sphere mixed with stearic acid. (C) Standard PE sphere and stearic acid mixture after ethanol rinse. Note: the images are the typical particles under each condition, respectively. They are not the same particles.

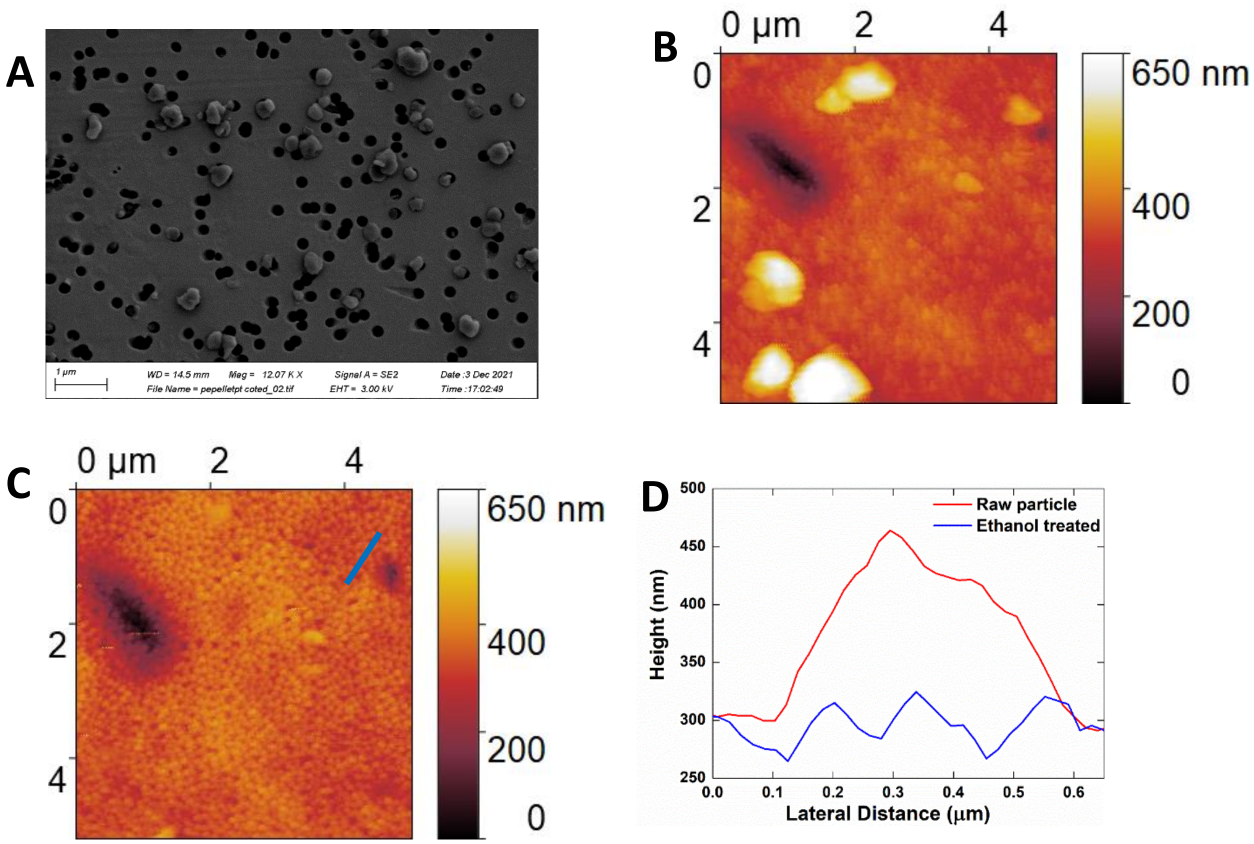

Figure S9. Nano-sized additives particles. (A) SEM image of typical raw nano particles captured by Au coated PC filter (pore size of 200 nm). (B) AFM image of typical raw nano particles captured by anodisc filter (pore size of 200 nm) and (C) the same spot treated by one drop of ethanol, respectively. The blue line indicates the crosssection direction. (D) Crosssection of the nano particles before and after ethanol treatment. Sample of LDPE pellets shaking in room temperature. Au coated PC filter was used to benefit SEM imaging while anodisc filter to benefit AFM imaging.

Table S1 summary of slip additives and PE.

Name	Formula	Melting point	Density	Solubility in Water	Solubility in Ethanol and Methanol
		°C	g/cm^3		
LDPE	$(C_2H_4)n$	104-115	0.88-0.96	insoluble	insoluble
HDPE	$(C_2H_4)n$	115-135	0.93-0.97	insoluble	insoluble
Behenamide	$C_{22}H_{45}NO$	110-113	0.865	insoluble	soluble
Stearamide	$C_{18}H_{37}NO$	102 - 104	0.868	insoluble	soluble
Erucamide	$C_{22}H_{43}NO$	77.5	0.874	insoluble	soluble
Oleamide	$C_{18}H_{35}NO$	70	0.879	insoluble	soluble
Stearic acid	$C_{18}H_{36}O_2$	69.3	0.941	insoluble	soluble
Erucic acid	$C_{22}H_{42}O_2$	33.8	0.86	insoluble	soluble

Melting point from Merck chemical datasheet. LDPE-low density PE. HDPE-high density PE.

Reference

1. Hahladakis, J. N.; Velis, C. A.; Weber, R.; Iacovidou, E.; Purnell, P., An overview of chemical additives present in plastics: Migration, release, fate and environmental impact during their use, disposal and recycling. *Journal of hazardous materials* **2018,** *344*, 179-199.
2. Manzano, E.; Rodriguez-Simón, L. R.; Navas, N.; Checa-Moreno, R.; Romero-Gámez, M.; Capitan-Vallvey, L. F., Study of the GC–MS determination of the palmitic–stearic acid ratio for the characterisation of drying oil in painting: La Encarnación by Alonso Cano as a case study. *Talanta* **2011,** *84*, (4), 1148-1154.
3. Calvo, B.; Cepeda, E. A., Solubilities of stearic acid in organic solvents and in azeotropic solvent mixtures. *Journal of Chemical & Engineering Data* **2008,** *53*, (3), 628-633.
4. Ralston, A.; Hoerr, C., The solubilities of the normal saturated fatty acids. *The Journal of Organic Chemistry* **1942,** *7*, (6), 546-555.
5. Heryanto, R.; Hasan, M.; Abdullah, E. C.; Kumoro, A. C., Solubility of stearic acid in various organic solvents and its prediction using non-ideal solution models. *ScienceAsia* **2007,** *33*, 469-472.
6. Noubigh, A., Stearic acid solubility in mixed solvents of (water+ ethanol) and (ethanol+ ethyl acetate): Experimental data and comparison among different thermodynamic models. *Journal of Molecular Liquids* **2019,** *296*, 112101.
